# Case series: effects of an induced molting protocol using levothyroxine in five captive banded penguins (genus *Spheniscus*)

**DOI:** 10.3389/fvets.2025.1544599

**Published:** 2025-09-10

**Authors:** Ji-Hyung Park, Seung-Hyun Seo, Sang Wha Kim, Yeong-Hun Kang

**Affiliations:** ^1^Laboratory of Aquatic Biomedicine, College of Veterinary Medicine and Institute of Veterinary Science, Kangwon National University, Chuncheon, Republic of Korea; ^2^Aqua Planet Yeosu, Yeosu-si, Republic of Korea; ^3^Biosafety Research Institute and Laboratory of Veterinary Dermatology, College of Veterinary Medicine, Jeonbuk National University, Iksan, Republic of Korea

**Keywords:** Humboldt penguins, African penguins, abnormal molt, forced-molt, levothyroxine

## Abstract

Penguin molts occur annually after breeding, allowing effective waterproofing and temperature regulation. However, aberrant molts frequently occur in penguins, which can lead to health problems if prolonged. Although the exact mechanisms underlying molting in birds remain unclear, some studies have focused on the roles of thyroid and sex hormones in penguins. Previous studies on forced molting using hormonal treatments have shown both success and failure. In particular, the use of synthetic thyroxine (levothyroxine) has mostly not been successful. This study aimed to induce molting by administering commercial levothyroxine to one Humboldt penguin (*Spheniscus humboldti*) and four African penguins (*Spheniscus demersus*) with abnormal molting, housed in aquaria. The planned levothyroxine dosing protocol was to start with 25 μg/kg PO q24h for 7 d, then increase it gradually to 50 μg/kg PO q24h for the next 7 d and 75 μg/kg PO q24h for 7 d, followed by a gradual decrease to 50 μg/kg PO q24h for 7 d, and 25 μg/kg PO q24h for the final 7 d. Three penguins received treatment according to this scheduled protocol, two of which initiated molting during the dosing period, while the third began to molt approximately 3 months post-treatment. Another penguin was administered the maximum dose for an additional week, which led to molting in the final week of treatment. In the remaining penguin, the medication was deemed to be insufficiently absorbed because of inadequate drug delivery methods. Consequently, the drug delivery strategy was changed during the 5^th^ week of treatment, and the treatment was extended for an additional 3 weeks beyond the original plan, after which the penguin began to molt. Therefore, all five penguins in this study exhibited molting following oral levothyroxine treatment, suggesting that this is a potential option for inducing molting in banded penguins (*Spheniscus* sp.) experiencing aberrant molt.

## 1 Introduction

Humboldt penguins (*Spheniscus humboldti*) and African penguins (*Spheniscus demersus*) belong to the genus *Spheniscus* and are also known as “banded penguins” due to the black band on their chest and the side of the body (1, 2). These medium-sized penguins weigh between 3 and 5 kg, with Humboldt penguins being slightly larger than African penguins (3). Humboldt penguins are distributed along the western coast of South America where the Humboldt current flows, while African penguins inhabit the southwestern coast of Africa (4, 5). In their natural habitats, Humboldt penguins are classified as vulnerable on the International Union for Conservation of Nature (IUCN) Red List. African penguins were uplisted from endangered to critically endangered in 2024 because of significant population declines (6, 7). These species, which are adapted to tropical to temperate climates, have a wide optimal temperature range, making them relatively suitable for keeping in captivity (8). Consequently, they are among the most observed penguins in zoos and aquariums in Korea.

Both African and Humboldt penguins breed year-round. During the breeding season, they lay one or two eggs and rear their chicks. Once chick care is no longer necessary, they undergo a post-nuptial molt (9, 10). Penguins molt annually. Humboldt penguins primarily molt during the austral summer, from January to March (11, 12). African penguins in Namibia typically molt during autumn (April–May) and summer (December), whereas those in South Africa molt during the summer months of November and December (13, 14). Zoologically-managed penguins in the Northern Hemisphere generally molt during the boreal summer, from June to August (15–17).

Penguin feathers are well adapted to aquatic environments. Their densely packed plumage provides excellent waterproofing and insulation properties (18, 19). Penguin feathers are generally categorized into contour feathers and downy feathers. In the case of Emperor penguins (*Aptenodytes forsteri*), afterfeathers and filoplumes were also identified (20). As feathers deteriorate over time, the annual molt—during which feathers are all replaced—is one of the major events in a penguin’s yearly cycle. Molting in birds is an energy-intensive process and, as such, does not overlap with reproduction or migration, which also require significant energy (18, 21–24). Penguin molts, in particular, are remarkably energy-demanding—catastrophic molts—unlike those of flying birds, as they involve the loss of all feathers over a short period (15, 19, 25). Consequently, penguins are extremely vulnerable to stress during this time (26, 27). In the pre-molt phase, penguins accumulate energy by consuming large quantities of food, increasing their body weight by up to 31% on average. During molting, all existing feathers are shed as new feathers grow beneath. Once molting begins, penguins cease food intake, and their body weight can decrease by up to 41% from the peak weight (15, 26). The feather-shedding period for African penguins averages 17–21 days (13, 15, 26, 28). Captive Humboldt penguins have demonstrated shorter molting periods (10 days), possibly because of more consistent food availability (2). In the post-molt phase, penguins return to the water to resume feeding and regain their body weight.

Abnormal molting is a frequent issue in penguins, with a prevalence rate of approximately 18.3% in populations under managed care (29), and is a particular concern in penguins kept in indoor environments (3). While the exact causes of abnormal molts have not been definitively identified, inappropriate light cycles, nutrition, and humidity levels are thought to contribute to the problem (8). In a previous study, abnormal molts were classified according to the timing and severity (Table 1) (30). Maintaining a proper molting cycle is crucial for the health of penguins.

**Table 1:** Grading scheme for abnormal molting (30).

Temporal grade	Description	Degree grade	Description
1.1	No molt observed on an annual interval	A	Full feather coverage but plumage may seem dull, brittle, or diminished
1.2	Penguin molted multiple times in a single year		
2	Prolonged molt: penguin experienced prolonged molting interval compared to average molting (17–21 days)	B	Partial molt, where <30% of plumage coverage is abnormal, including absence of feathers or poor feather quality
3	Arrested molt: penguin began a molt but failed to complete the process for the entirety of its feathers	C	Severe abnormal molt, where >30% of plumage is abnormal, including absence of feathers or poor feather quality

The precise mechanisms underlying avian molting have not been fully elucidated (31); however, some studies on penguins have suggested that molting is closely associated with sex and thyroid hormones. Studies have shown that in Humboldt, King (*Aptenodytes patagonicus*), Adelie (*Pygoscelis adeliae*), and Emperor penguins, sex hormone levels (testosterone, estradiol) increase during the breeding season, followed by gonadal involution (16, 32–35). When steroid hormone levels reduce to baseline, the plasma concentration of thyroxine (T4) begins to rise and rapidly surges to its peak, coinciding with the onset of molting. In avian species, sex hormones may play a role in suppressing T4 levels (16, 31). Molting continues until thyroid hormone levels decline, at which point it ceases. Subsequently, sex hormone levels increase again.

Several cases of hormone-induced molting in penguins have been reported. In Chinstrap penguins (*Pygoscelis antarcticus*), molt could be induced by administering 30 mg/kg of medroxyprogesterone through five sequential weekly injections, followed by a repetition of the same regimen a month later (36). Successful cases of molt induction using subcutaneous implants of 5.4 mg melatonin have also been reported (30). In juvenile Yellow-eyed penguins (*Megadyptes antipodes*), molt was induced by feeding 10 g/kg of fresh beef thyroid gland once daily for approximately 18–26 days (37). In other bird species, exogenous thyroxine has been used to induce molting (e.g., in turkeys and chickens) (22, 38).

Levothyroxine is a synthetic thyroxine (T4) hormone used to treat hypothyroidism in humans and other animals (39, 40). However, to authors’ knowledge, no previous study has effectively induced molting in penguins by using levothyroxine (30, 37). Therefore, this study aimed to explore a method for administering T4, which is known to be associated with avian molting, by using a commercially available medication. This study presents a novel approach for inducing molting in penguins by gradually applying levothyroxine to Humboldt and African penguins that exhibited molting abnormalities.

## 2 Case descriptions

Aquaria A and B collectively housed 30 penguins in indoor enclosures. Aquarium A maintained a mixed colony of 13 penguins, comprising eight Humboldt penguins (four females and four males) and five African penguins (three females and two males). The exhibition environment was managed at an air temperature of 21.25 ± 3.03°C and a water temperature of 18.71 ± 0.73°C. Aquarium B exclusively housed 17 African penguins (8 females, 9 males) in an environment with an air temperature of 19.25 ± 2.65°C and a water temperature of 16.3 ± 1.8°C. Both facilities fed the penguins thawed whole Korean sandlance and capelin twice daily. Additionally, they supplied the penguins with 1–2 tablets of a multivitamin supplement (5 M25, Mazuri Exotic Animal Nutrition, St. Louis, MO, USA) daily. The terrestrial enclosure undergoes daily cleaning protocol, and semiannual sedimentation plate testing is conducted to assess airborne fungal contamination within the enclosure environment.

Between 2019 and 2022, one Humboldt penguin and four African penguins showed symptoms of abnormal molting. Among these, four individuals did not undergo natural molting even 1 year after their last molt, with the alopecia area exceeding 30% of their body surface (grade C). This condition was deemed severe enough to warrant forced molting. The other individual maintained waterproofing abilities but had not molted for 2 years and 10 months, leading to a decision to initiate treatment. Detailed information on the penguins treated in this study is provided in Table 2. Macroscopic examination of each penguin revealed no clinical signs of infection, including erythema, papules, pustules, or crusting lesions. Photographic documentation was obtained from both dorsal and ventral views to quantify the extent of lesional involvement.

**Table 2:** Case description of five penguins with abnormal molting in the present study. (ND = not detectable).

Parameters	Case 1	Case 2	Case 3	Case 4	Case 5
Penguin name	P1	P2	P3	P4	P5
Species	Humboldt penguin	African penguin			
Location	Aquarium A	Aquarium B	Aquarium B	Aquarium B	Aquarium A
Sex	Female	Female	Male	Female	Female
Age of onset	6 yr	6 yr	15 yr	11 yr	8 yr
Last molt before treatment	1 yr. 1 mo	2 yr. 5 mo	2 yr. 10 mo	4 yr. 5 mo	2 yr. 6 mo
Lesions of body surface %	60%	40%	10%	70%	50%
Abnormal molting Grades	1.1C	1.1C	1.1A	1.1C	1.1C
Before treatment	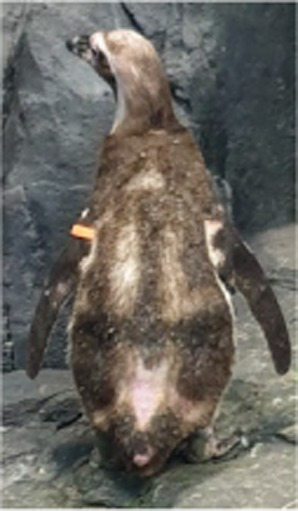	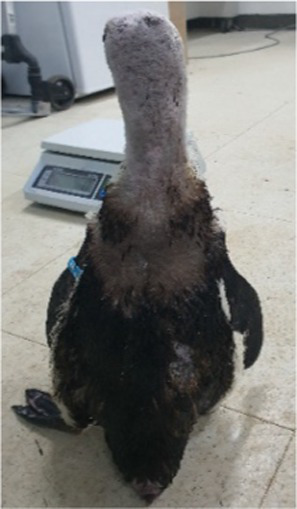	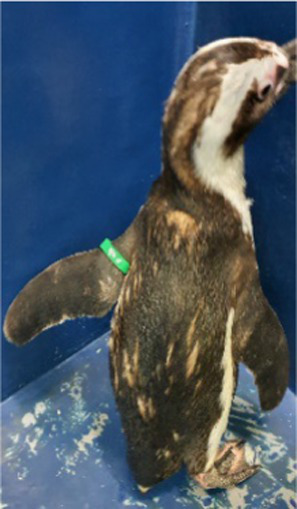	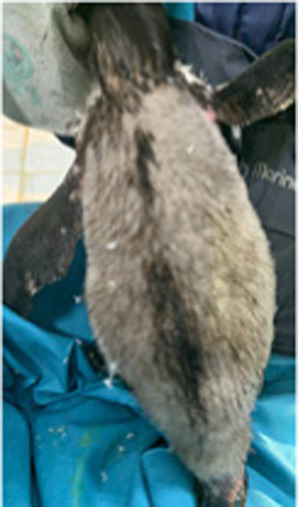	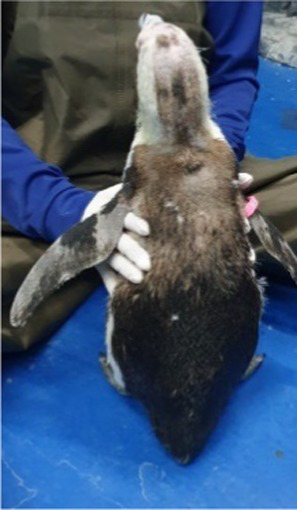
During molting	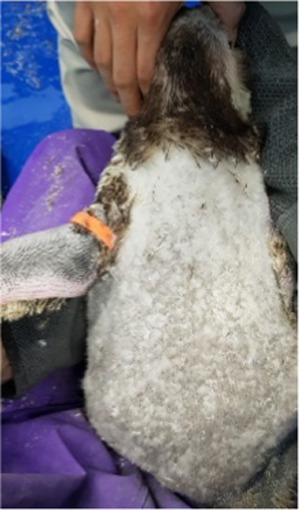	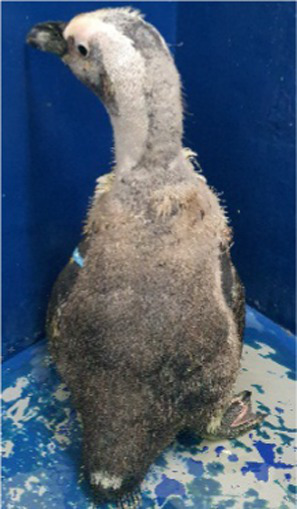	ND	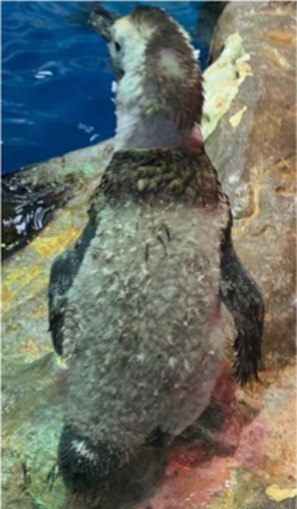	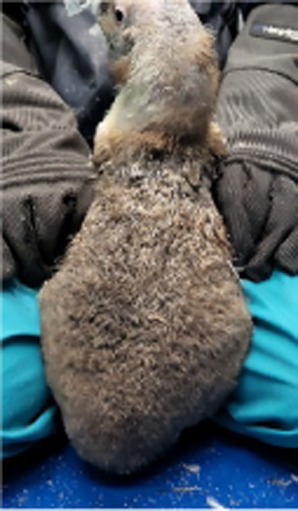
After treatment	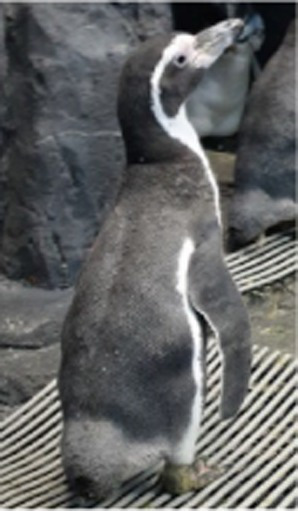	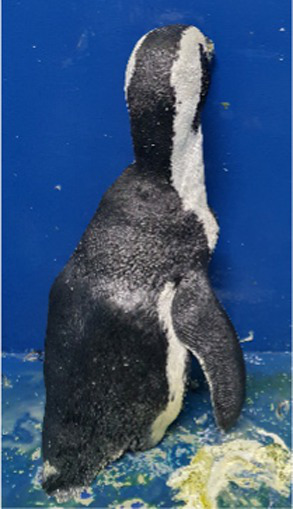	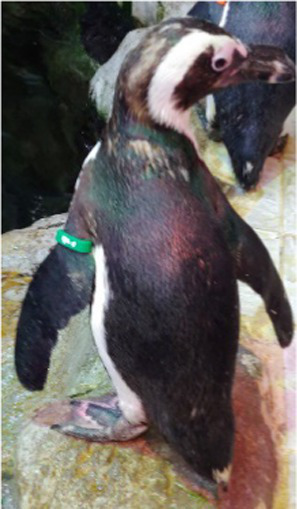	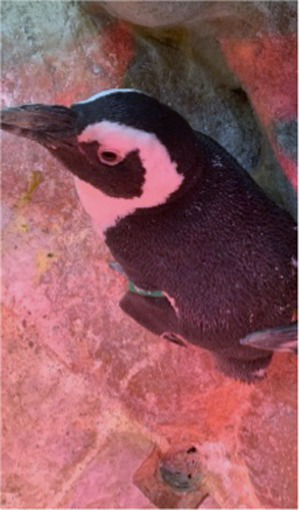	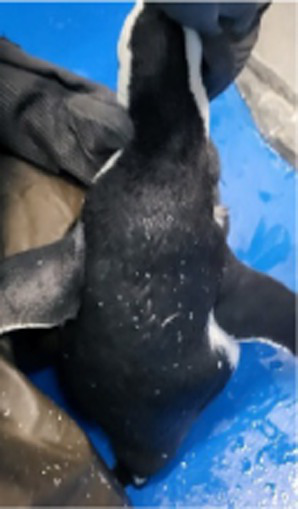

The molt-inducement treatment protocol was established by referencing methods used for inducing molt in raptors (41). Levothyroxine (25 μg tablets, Dalim Biotech, Gangwon, South Korea) was administered orally once daily, by placing the tablets in the fish fed at mealtime, and the dose was adjusted weekly. The specific dosage schedule was 25 μg/kg PO q24h for 7 d, 50 μg/kg PO q24h for 7 d, 75 μg/kg PO q24h for 7 d, 50 μg/kg PO q24h for 7 d, and 25 μg/kg PO q24h for 7 d, i.e., a gradual increase followed by a decrease. The timing of the molt was determined visually as the period between the first observation of continuous shedding of old feathers and the observed replacement of all new feathers.

### 2.1 Case 1

Penguin 1 (P1) was a female Humboldt penguin with a history of molting annually. In 2018, at approximately 5 years of age, the subject developed focal alopecia in the mid-dorsal region of the tail area. Thereafter, molt initiation was observed, characterized by chest plumage ruffling with concurrent minor feather loss; however, molting was temporarily suspended for 2 weeks, after which it resumed as comprehensive whole-body molting. In 2019, P1 exhibited similar tail region symptoms without concurrent molting signs. The affected area gradually expanded, leading to symmetrical feather loss across the dorsal surface. The alopecia then spread to the head and flippers, and the chest feathers appeared puffy. Dermatological examination of the alopecia regions demonstrated an absence of infectious manifestations, including erythema, papules, and pustular formations. Microscopic analysis via sticky tape sampling revealed no detectable bacterial or fungal organisms. Furthermore, fungal culture results obtained through feather plucking yielded negative findings, effectively ruling out fungal infection. Given that the initial alopecia lesions were observed near the tail region, impression cytology of the uropygial gland was conducted, revealing the presence of coccoid bacteria. Consequently, oral enrofloxacin therapy was initiated; however, clinical improvement was not observed following antibiotic treatment. Based on these findings, the lesions were considered to have a non-infectious etiology; therefore, hormonal therapy was initiated as a therapeutic trial.

To induce molting, levothyroxine was administered by embedding the tablets in fish during feeding times, with dosage gradually increased according to protocol and subsequently tapered. Molting commenced during the 3^rd^ week of treatment, evidenced by initial feather shedding. Feather replacement occurred sequentially, beginning with the trunk region, followed by the head and neck areas, with completion achieved by the 4^th^ week. Serum biochemical analysis performed 2 weeks post-treatment showed no significant findings in hepatic, renal, or electrolyte profiles.

### 2.2 Case 2

Penguin 2 (P2) was a female African penguin with an established pair bond. Following the initial molt, P2 underwent a second molt 1 year later; however, subsequent molting cycles occurred at extended intervals of 15 months and 18 months, respectively. After successfully raising a chick in 2021, P2 failed to molt and produced one infertile egg in 2022. Coinciding with this period, P2 experienced progressive feather loss, primarily affecting the upper part of the body. Severe alopecia was observed on the head and neck, while the upper back, chest and flippers exhibited coarse and sparse feathering. Treatment was initiated in 2022, approximately 2 years and 5 months following the last documented molt. Dermatological examination revealed no evidence of infection. Following the established protocol, levothyroxine was administered for 5 weeks. Molting started during the 2^nd^ week of treatment with generalized feather loss and concluded by the 3^rd^ week with the emergence of healthy new plumage. Although molting was completed, levothyroxine tapering continued through the 5^th^ week of treatment.

### 2.3 Case 3

Penguin 3 (P3) was a male African penguin. The animal exhibited no significant health issues and typically underwent annual molting cycles until 2014. By late 2017, slight fluffing and minor feather loss were observed in a small area. This occurred 2 months before the catastrophic molt progressed over 20 days. In 2019, P3 exhibited focal feather loss in the neck regionAdministering omega-3 and omega-6 fatty acids supplements (Coatex, VetPlus, Lytham St. Annes, Lancashire, England) yielded limited results. Over time, multifocal lesions developed around the neck and dorsal areas, although they involved only 10% of the body surface.

Levothyroxine treatment was initiated according to the abovementioned protocol at 2 years and 10 months after the last molt. Some improvement in dorsal part was noted by the end of the 5-week treatment period, although catastrophic molting did not occur. However, 112 days after completing treatment, a catastrophic molt was observed that lasted 13 days.

### 2.4 Case 4

Penguin 4 (P4) was a female African penguin and the mate of P3. P4 laid one to two eggs annually but did not lay any eggs from 2016 until 2019. This animal typically underwent molting every 2 years, with the last molt occurring in 2015. In 2017, some feathers began to fluff, and progressive alopecia was observed. Alopecia mostly affected the back and flippers, with the dorsal contour feathers shedding, except in the midline, leaving only downy feathers. Intermittent nutritional supplements with MyBeau Vet Collections Skin & Hair (palaMOUNTAINS, Wanganui, New Zealand) and Coatex were administered but resulted in limited improvement. Cytological examination of uropygial gland impression smears revealed abundant yeast organisms, prompting initiation of topical ketoconazole shampoo therapy. However, the subject exhibited emetic episodes during the second treatment application, necessitating immediate discontinuation of the therapeutic regimen. Consequently, this treatment approach yielded minimal therapeutic benefit.

**Figure 1:**
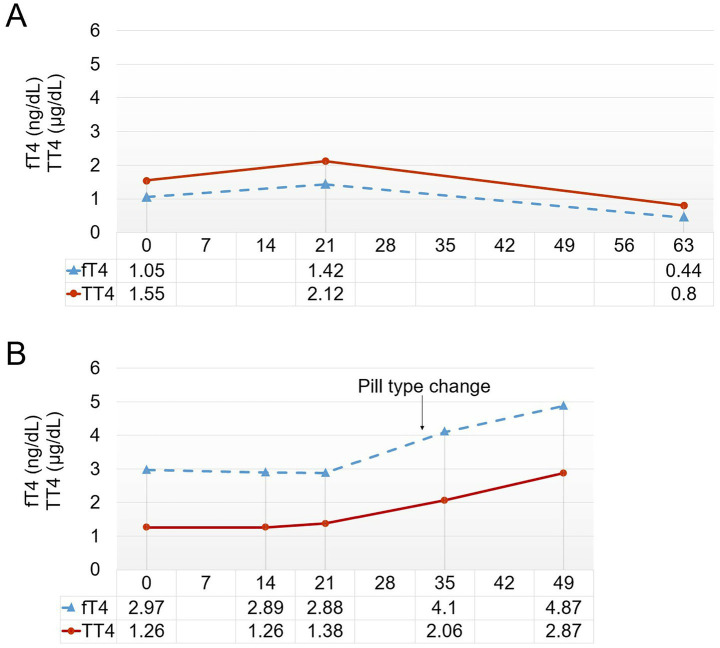
**(A)** Thyroid hormone levels of Penguin 4. 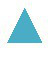: free thyroxine (fT4), 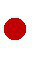: total thyroxine (TT4). **(B)** Thyroid hormone levels of Penguin 5. On day 33, the method was changed to administering levothyroxine tablets directly via fish, without first including the tablet pieces in hard gelatin capsules (arrow).

Treatment with levothyroxine was initiated 4 years and 5 months after the last molt, and despite a 3-week course of treatment, no signs of molting were observed. We decided to provide an additional 1 week of treatment at the maximum dosage (75 μg/kg), and then started tapering (total of 6 weeks of treatment). From the start of the 6^th^ week, at a dosage of 25 μg/kg, the feathers began to shed. By the end of the treatment, significant feather loss occurred in the flippers, and molting was completed approximately 1 week later. Thyroid hormone levels were assessed, showing temporary increases in total T4 (TT4) and free T4 (fT4) levels during treatment, followed by a decrease in all measured thyroid hormone levels post-treatment (Figure 1A).

### 2.5 Case 5

Penguin 5 (P5) was a female African penguin without a mate and was introduced from Aquarium B to Aquarium A in 2019. After the transfer, P5 did not experience normal molting; instead, starting in 2021, it began to develop a gradual feather-shedding condition that progressively affected the back of the head, neck, flipper, and upper back, primarily impacting the upper body.

In 2022, levothyroxine treatment was initiated, but no signs of molting were observed until the 5^th^ week. Thyroid hormone testing during treatment revealed no increase, with levels remaining essentially steady. A significant difference from previous cases was that the medication was not administered in tablet form per se; rather, tablet pieces were encapsulated in 500-mg hard gelatin capsules. This was suspected to hinder the digestion and absorption of the drug in the penguin’s gastrointestinal tract. Consequently, on the 33^rd^ day of treatment, a switch was made to the tablet form. Following this change, a hormone level test on the 35^th^ day indicated an increase in both TT4 and fT4 (Figure 1B). Given these absorption issues, the treatment was extended to induce molting. Observing the response to the lowest dosage of 25 μg/kg, molting began on the 45^th^ day of treatment and was completed by the 56^th^ day. The treatment was continued for a total of 8 weeks until molting was fully achieved.

## 3 Discussion

In this study, we devised a protocol for inducing molting in penguins by oral levothyroxine treatment. Successful molt induction in five aquarium-housed penguins using this novel approach indicated that this approach may be an option for inducing molting in banded penguins (*Spheniscus* sp.) with molting abnormalities.

The dense plumage of penguins provides waterproofing and insulation, which are crucial for swimming. Inadequate molting can lead to difficulties in thermoregulation and may adversely affect metabolism (42). One study showed that penguins with molting abnormalities have a lower survival rate, and arrested molts can render them vulnerable to diseases, such as aspergillosis and avian malaria (26–28). Additionally, these penguins are at risk of developing pododermatitis due to prolonged stay on land. Ignoring molting issues can also negatively impact esthetics, leading to perceptions of poor management among zoo and aquarium visitors (30). For these reasons, inducing molting is essential for the health of penguins.

**Figure 2:**
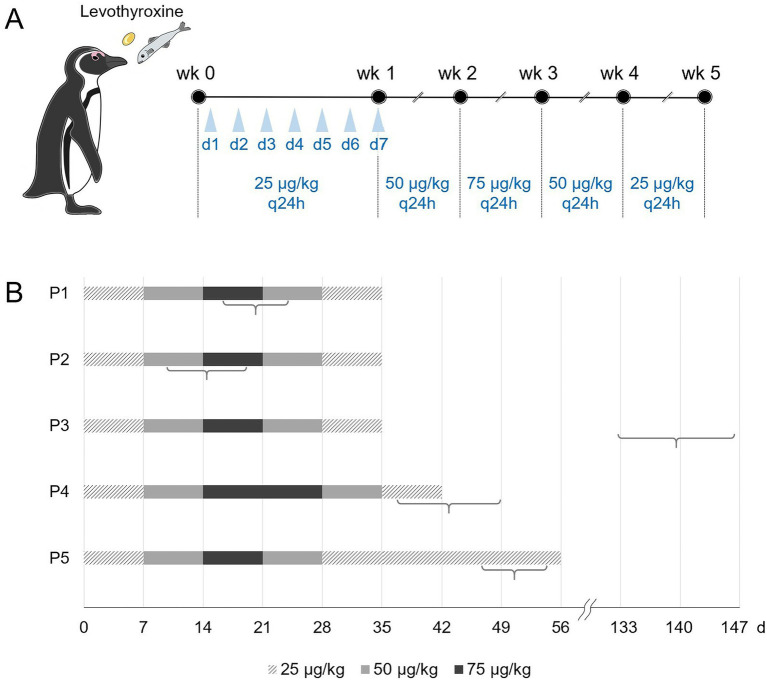
Levothyroxine treatment for inducing molt in banded penguins. **(A)** Planned treatment protocol. The drug concentration is increased from 25 to 50 μg/kg and then to 75 μg/kg weekly. Subsequently, the drug concentration is decreased to 50 μg/kg and then to 25 μg/kg weekly. Levothyroxine is administered to penguins through fish containing the medicine. **(B)** Actual levothyroxine dosing schedule used in Penguin 1 (P1) to Penguin 5 (P5). Individual molting periods are noted in parentheses.

The mechanisms underlying molting are complex and not fully understood; however, various hormonal factors involved in molting are under investigation (31). Previous studies have successfully induced molting in Yellow-eyed penguins by feeding them thyroid glands (37). Thyroid glands synthesize and secrete two major hormones: T4 and triiodothyronine (T3). T4 appears to be more important than T3 for feather growth and molting in birds (16, 43). This study aimed to advance the abovementioned approach by using a commercial drug, synthetic T4, to induce molting and yielded meaningful results across all five cases. However, treatment adjustments were necessary for each case, and the timing of molting varied slightly (Figure 2): P1, P2, and P3 followed the planned protocols, whereas P4 and P5 required extended treatment.

In the case of P3, only minor improvements were noted in dorsal feathers after 5 weeks of treatment. A catastrophic molt was observed 3 months after the end of treatment. While it remains unclear whether the treatment directly triggered molting or if it was coincidental, it is plausible that the recent medication acted as a trigger after nearly 3 years without molting. Additionally, the success or failure of treatment may correlate with the timing of medical intervention. Molting in P1, P2, P4, and P5 were classified as grade C—a severe condition—and exhibited favorable treatment responses, whereas P3 was deemed to be grade A, with only minor lesions affecting the condition of 10% of the feathers. The severity of molting likely influences treatment response, indicating the need for further investigation.

In case 4, following 3 weeks of treatment, TT4 levels were 2.12 μg/dl, and fT4 levels were 1.42 ng/dl, which appeared insufficient to induce molting. According to published literature, typical T4 levels in Humboldt penguins range from 1.0 to 2.5 μg/dl, with a tendency to surge to 3.0–4.0 μg/dl during the molting period (16). Although these values are not species-specific for African penguins, it seems that a threshold hormone concentration must be reached to induce molting in penguins. Consequently, while continuous blood monitoring was not performed, the extended treatment duration likely enabled TT4 and fT4 levels in P4 to reach potentially sufficient concentrations, ultimately resulting in successful molting induction. In this investigation, we prioritized minimizing animal stress by employing minimal physical restraint procedures. Therefore, we implemented a protocol whereby one representative penguin from each facility underwent blood sampling at three time points: treatment initiation, mid-treatment, and treatment completion, exclusively for thyroid hormone level assessment. Further research is warranted, particularly regarding periodic blood tests, to ascertain threshold levels of TT4 and fT4 necessary for molt induction and to monitor overall health status. Future studies should document thyroid hormone levels in African penguins with normal molting patterns to establish reference values for treatment. Moreover, comparing hormone levels in penguins housed exclusively indoors with those kept outdoors under natural UVB exposure and light cycles may provide further insight into environmental factors influencing molting physiology.

Levothyroxine undergoes disintegration and dissolution processes in the gastric environment, which are necessary for its absorption in the small intestine (44). In the case of P5, the lack of molting by the 5^th^ week and the absence of increased thyroid hormone levels may have been significantly influenced by the method of drug administration. Unlike other cases where medication was administered in tablet form, treatment of P5 involved encapsulating tablet fragments within hard gelatin capsules to achieve precise weight-based dosing, thus requiring a two-step disintegration process. Hard capsules are made from animal gelatin derived from the skin or bones of cattle and pigs, and their dissolution is influenced by gastric acidity (45). One previous study showed that the dissolution rate of hard gelatin capsules occurs more slowly at pH 2–3 than at pH 1.2 (46). The average gastric pH in African penguins is approximately 2.75, while that of Magellanic (*Spheniscus magellanicus*) and Gentoo (*Pygoscelis papua*) penguins is 2.5 and 2.3, respectively (47, 48). This indicates that hard gelatin capsules may require additional time to dissolve. Moreover, gastrointestinal absorption is closely related to transit time (49). Although many variables must be considered, food transit in birds is generally faster than that in mammals and other vertebrates (50, 51). Furthermore, levothyroxine is recommended to be administered directly, without food, as food can hinder absorption (52, 53). However, administering the medication solely in pill form in penguins is impractical, necessitating its combination with fish, although this may have further reduced absorption rates. Given the relatively high gastric pH, short transit times, and requirement to administer medication with food, it is crucial to minimize factors that could interfere with drug absorption. In P5, an increase in thyroid hormone levels was observed after omitting the hard gelatin capsules and administering tablets directly via fish feeding from the 33^rd^ day of treatment.

From 2019 to 2024, five of the 13 penguins (4 females, 1 male) at Aquarium A and four of the 17 penguins (2 females, 2 males) at Aquarium B exhibited molting problems. This equated to prevalence rates of 41.7 and 23.5%, respectively, which were higher than the previously reported rate of 18.3%, suggesting a potential correlation of aberrant molting with indoor enclosure conditions. Improving modifiable environmental factors, such as photoperiod regulation, UV light exposure, and offering diverse diets, could prove beneficial (8).

Furthermore, the higher incidence of issues among females (66.7%) in this study is noteworthy, as previous studies indicated a slightly higher incidence among males (30). Based on observations of female penguins in this study, we found a potential correlation between cessation of egg-laying and abnormal molting patterns. Notably, in case 4, P4 exhibited an absence of egg-laying prior to the onset of the molting abnormality. Previous research has demonstrated that thyroidectomy in egg-laying turkeys resulted in suspended egg production, which was subsequently restored through T4 supplementation, indicating the essential role of thyroid hormones in egg production (22). These findings suggest that disrupted egg-laying patterns may serve as early indicators of impending molt abnormalities, underscoring the importance of future research investigating the relationship between egg-laying frequency and thyroid hormone levels in penguins who display irregular molting behavior.

To date, only one of the treated penguins described in this report has demonstrated recovery of normal annual molting patterns. This animal, P2, initiated egg-laying approximately 4 months post-treatment and underwent molting in the subsequent year. The duration of molting was 12 days. Since then, P2 has maintained its molting and egg-laying patterns every year. Except for P2, the other penguins exhibited recurrent molting abnormalities. P1 initially regained its annual egg-laying behavior and normal molting pattern after pairing with a new mate. However, in 2024, this individual again experienced abnormal feather loss beginning in March, followed by a prolonged molting period that extended until August. P3 has not undergone molting for approximately 3 years and 7 months since its last forced molt. P4 showed a sharp rise in estradiol levels in the blood immediately after the forced molt and started laying eggs immediately. However, P4 molted again approximately 2 years after the forced molt ended. P5 has not molted for two and a half years, but has again demonstrated gradual feather loss starting from the back in 2024. Forced molting is not a fundamental solution for molting abnormalities. Although levothyroxine is generally considered safe, repeated treatments may result in adverse effects associated with hyperthyroidism, including elevated heart rate, increased body temperature, excitable hyperkinetic behavior, and panting (54). For indoor-bred penguins, optimization of environmental factors is critical prior to initiating pharmacological intervention. Recently, aquaria A and B have implemented photoperiod adjustments to align with seasonal variations in Korea.

In conclusion, our study suggests that when molting disorders in penguins persist despite environmental optimization, medical intervention with levothyroxine therapy is recommended. The dosage protocol is 25 μg/kg PO q24h for 7 d, 50 μg/kg PO q24h for 7 d, 75 μg/kg PO q24h for 7 d, 50 μg/kg PO q24h for 7 d, and 25 μg/kg PO q24h for 7 d. These findings have significant implications for the future management and conservation of penguin populations.

## Data Availability

The original contributions presented in the study are included in the article/Supplementary material, further inquiries can be directed to the corresponding authors.

## References

[1] Crawford RJM Kemper J Underhill LG. African penguin: (*Spheniscus demersus*) In: Borboroglu PG Boersma PD, editors. Penguins: Natural history and conservation. Seattle: University of Washington Press (2013). 211–31.

[2] De la Puente S Bussalleu A Cardeña M Valdés-Velasquez A Majluf P Simeone A. Humboldt penguin (*Spheniscus humboldti*) In: Borboroglu PG Boersma PD, editors. Penguins: Natural history and conservation. Seattle: University of Washington Press (2013). 265–83.

[3] Association of Zoos and Aquariums Penguin Taxon Advisory Group. Penguin husbandry manual. 3rd ed. Silver Spring (MD): Association of Zoos and Aquariums (2005). 141 p.

[4] León F Pizarro E Noll D Pertierra LR Parker P Espinaze MPA. Comparative genomics supports ecologically induced selection as a putative driver of banded penguin diversification. Mol Biol Evol. (2024) 41:msae166. doi: 10.1093/molbev/msae166, PMID: 39150953 PMC11371425

[5] Wilson RP Duffy DC Wilson M-P Araya B. Aspects of the ecology of species replacement in Humboldt and Magellanic penguins in Chile. Gerfaut. (1995) 85:49–61.

[6] BirdLife International. (2024). *Spheniscus demersus*. The IUCN red list of threatened species e.T22697810A256021744 [Accessed November 29, 2024].

[7] BirdLife International. (2020). *Spheniscus humboldti*. The IUCN red list of threatened species 10.2305/IUCN.UK.2020-3.RLTS.T22697817A182714418.en

[8] Association of Zoos and Aquariums Penguin Taxon Advisory Group. Penguin (Spheniscidae) care manual. Silver Spring (MD): Association of Zoos and Aquariums (2014). 143 p.

[9] Culik BM Luna-Jorquera G. Satellite tracking of Humboldt penguins (*Spheniscus humboldti*) in northern Chile. Mar Biol. (1997) 128:547–56. doi: 10.1007/s002270050120

[10] Whittington PA Randall RM Randall BM Wolfaardt AC Crawford RJM Klages NTW. Patterns of movements of the African penguin in South Africa and Namibia. Afr J Mar Sci. (2005) 27:215–29. doi: 10.2989/18142320509504080

[11] Paredes R Zavalaga CB Boness DJ. Patterns of egg laying and breeding success in Humboldt penguins (*Spheniscus humboldti*) at Punta San Juan, Peru. Auk. (2002) 119:244–50. doi: 10.1093/auk/119.1.244

[12] Simeone A Araya B Bernal M Diebold EN Grzybowski K Michaels M. Oceanographic and climatic factors influencing breeding and colony attendance patterns of Humboldt penguins *Spheniscus humboldti* in Central Chile. Mar Ecol Prog Ser. (2002) 227:43–50. doi: 10.3354/meps227043

[13] Crawford RJM Hemming M Kemper J Klages NTW Randall RM. S24-2 molt of the African penguin, *Spheniscus demersus*, in relation to its breeding season and food availability. Acta Zool Sin. (2006) 52:444–7.

[14] Waller LJ. (2011). The African penguin *Spheniscus demersus*: Conservation and management issues. PhD thesis. Cape Town: University of Cape Town.

[15] Bennett K. Molt patterns of black-footed penguins (*Spheniscus demersus*) at Baltimore zoo. Spheniscus Penguin Newsl. (1991) 4:1–4.

[16] Otsuka R Machida T Wada M. Hormonal correlations at transition from reproduction to molting in an annual life cycle of Humboldt penguins (*Spheniscus humboldti*). Gen Comp Endocrinol. (2004) 135:175–85. doi: 10.1016/j.ygcen.2003.09.007, PMID: 14697303

[17] Scholten CJ. Breeding biology of the Humboldt penguin *Spheniscus humboldti* at Emmen zoo. Int Zoo Yearb. (1987) 26:198–204. doi: 10.1111/j.1748-1090.1987.tb03158.x

[18] Payne RB. Mechanisms and control of molt In: Farner DS King JR, editors. Avian biology volume 2. New York, NY: Academic Press (1972). 103–55.

[19] Stonehouse B. The general biology and thermal balance of penguins In: Cragg JB, editor. Advances in ecological research, vol. 4. London: Academic Press (1967). 131–96.

[20] Williams CL Hagelin JC Kooyman GL. Hidden keys to survival: the type, density, pattern and functional role of emperor penguin body feathers. Proc R Soc B. (2015) 282:20152033. doi: 10.1098/rspb.2015.2033, PMID: 26490794 PMC4633883

[21] Gwinner E. Circannual rhythms in birds. Curr Opin Neurobiol. (2003) 13:770–8. doi: 10.1016/j.conb.2003.10.010, PMID: 14662381

[22] Lien RJ Siopes TD. Effects of thyroidectomy on egg production, molt, and plasma thyroid hormone concentrations of Turkey hens. Poult Sci. (1989) 68:1126–32. doi: 10.3382/ps.0681126, PMID: 2780487

[23] Swaddle JP Witter MS. The effects of molt on the flight performance, body mass, and behavior of European starlings (*Sturnus vulgaris*): an experimental approach. Can J Zool. (1997) 75:1135–46. doi: 10.1139/z97-136

[24] Lindström Å Visser GH Daan S. The energetic cost of feather synthesis is proportional to basal metabolic rate. Physiol Zool. (1993) 66:490–510. doi: 10.1086/physzool.66.4.30163805

[25] Kooyman GL Hunke EC Ackley SF van Dam RP Robertson G. Moult of the emperor penguins: travel, location and habitat selection. Mar Ecol Prog Ser. (2000) 204:269–77. doi: 10.3354/meps204269

[26] Cooper J. Moult of the black-footed penguin. Int Zoo Yearb. (1978) 18:22–7. doi: 10.1111/j.1748-1090.1978.tb00211.x

[27] Davis DG. Keeping penguins in captivity: the penguin paradox. Int Zoo Yearb. (1967) 7:3–11. doi: 10.1111/j.1748-1090.1967.tb00290.x

[28] Parsons NJ Vanstreels RET Schaefer AM. Prognostic indicators of rehabilitation outcomes for adult African penguins (*Spheniscus demersus*). J Wildl Dis. (2018) 54:54–65. doi: 10.7589/2017-06-146, PMID: 29059011

[29] Randall RM Randall BM Cooper J Frost PGH. A new census method for penguins tested on jackass penguins *Spheniscus demersus*. Ostrich. (1986) 57:211–5. doi: 10.1080/00306525.1986.9633658

[30] Golembeski M Sander SJ Kottyan J Sander WE Bronson E. Factors affecting abnormal molting in the managed african penguin (*Spheniscus demersus*) population in North America. J Zoo Wildl Med. (2020) 50:917–26. doi: 10.1638/2019-0080, PMID: 31926524

[31] Dawson A. Avian molting In: Scanes CG, editor. Sturkie’s avian physiology. 6th ed. Amsterdam: Academic Press (2014). 907–17. doi: 10.1016/B978-0-12-407160-5.00038-5

[32] Cherel Y Leloup J Le Maho Y. Fasting in king penguin. Part II. Hormonal and metabolic changes during molt. Am J Phys. (1988) 254:R178–84. doi: 10.1152/ajpregu.1988.254.2.R178, PMID: 3278625

[33] Groscolas R Jallageas M Goldsmith A Assenmacher I. The endocrine control of reproduction and molt in male and female emperor (*Aptenodytes forsteri*) and adelie (*Pygoscelis adeliae*) penguins. i. Annual changes in plasma levels of gonadal steroids and LH. Gen Comp Endocrinol. (1986) 62:43–53. doi: 10.1016/0016-6480(86)90092-4, PMID: 3781216

[34] Groscolas R Leloup J. The endocrine control of reproduction and molt in male and female emperor (*Aptenodytes forsteri*) and adelie (*Pygoscelis adeliae*) penguins. II. Annual changes in plasma levels of thyroxine and triiodothyronine. Gen Comp Endocrinol. (1986) 63:264–74. doi: 10.1016/0016-6480(86)90164-4, PMID: 3781233

[35] Mauget R Jouventin P Lacroix A Ishii S. Plasma LH and steroid hormones in King penguin (*Aptenodytes patagonicus*) during the onset of the breeding cycle. Gen Comp Endocrinol. (1994) 93:36–43. doi: 10.1006/gcen.1994.1005, PMID: 8138117

[36] Reidarson TH McBain JF Denton D. The use of medroxyprogesterone acetate to induce molting in chinstrap penguins (*Pygoscelis antarctica*). J Zoo Wildl Med. (1999) 30:278–80. PMID: 10484146

[37] Webster RK Aguilar RF Argandona-Gonzalez AK Conayne P De Sousa D Sriram A. Forced molt in four juvenile yellow-eyed penguins (*Megadyptes antipodes*). J Wildl Dis. (2016) 52:809–16. doi: 10.7589/2015-11-305, PMID: 27505039

[38] Sekimoto K Imai K Suzuki M Takikawa H Hoshino N Totsuka K. Thyroxine-induced molting and gonadal function of laying hens. Poult Sci. (1987) 66:752–6. doi: 10.3382/ps.0660752, PMID: 3615336

[39] Plumb DC. Plumb’s veterinary drug handbook, 6th ed. Vancouver: Blackwell Publishing (2008). p. 534–535.

[40] Sarfaraz NK (2004). Handbook of pharmaceutical manufacturing formulations: compressed solid products Boca Raton, FL CRC Press. 151–152.

[41] Carpenter JW. Exotic Animal Formulary. 5th ed. St. Louis(MO): Saunders (2005). 240 p.

[42] Green JA Butler PJ Woakes AJ Boyd IL. Energetics of the moult fast in macaroni penguins *Eudyptes chrysolophus*. J Avian Biol. (2004) 35:153–61. doi: 10.1111/j.0908-8857.2004.03138.x

[43] Queen WH Christensen VL May JD. Supplemental thyroid hormones and molting in Turkey breeder hens. Poult Sci. (1997) 76:887–93. doi: 10.1093/ps/76.6.887, PMID: 9181624

[44] Virili C Antonelli A Santaguida MG Benvenga S Centanni M. Gastrointestinal malabsorption of thyroxine. Endocr Rev. (2019) 40:118–36. doi: 10.1210/er.2018-00168, PMID: 30476027

[45] Duconseille A Astruc T Quintana N Meersman F Sante-Lhoutellier V. Gelatin structure and composition linked to hard capsule dissolution: A review. Food Hydrocolloids. (2015) 43:360–76. doi: 10.1016/j.foodhyd.2014.06.006

[46] Muhammad NA Newton JM. The influence of pH of dissolution fluid and particle size of drug on the in-vitro release of drug from hard gelatin capsules. J Pharm Pharmacol. (1983) 35:345–9. doi: 10.1111/j.2042-7158.1983.tb02954.x, PMID: 6135771

[47] Beasley DE Koltz AM Lambert JE Fierer N Dunn RR. The evolution of stomach acidity and its relevance to the human microbiome. PLoS One. (2015) 10:e0134116. doi: 10.1371/journal.pone.0134116, PMID: 26222383 PMC4519257

[48] Fujimori S. Gastric acid level of humans must decrease in the future. World J Gastroenterol. (2020) 26:6706–9. doi: 10.3748/wjg.v26.i43.6706, PMID: 33268958 PMC7684463

[49] Kimura T Higaki K. Gastrointestinal transit and drug absorption. Biol Pharm Bull. (2002) 25:149–64. doi: 10.1248/bpb.25.149, PMID: 11853157

[50] Clench MH Mathias JR. Intestinal transit: how can it be delayed long enough for birds to act as long-distance dispersal agents? Auk. (1992) 109:933–6. doi: 10.2307/4088179

[51] Kline S Kottyan J Phillips J Wack A Pate N Bronson E. The radiographic and endoscopic anatomy and digestive mechanisms of captive African penguins (*Spheniscus demersus*). J Zoo Wildl Med. (2020) 51:371–8. doi: 10.1638/2019-0076, PMID: 32549567

[52] Benvenga S Bartolone L Squadrito S Lo Giudice F Trimarchi F. Delayed intestinal absorption of levothyroxine. Thyroid. (1995) 5:249–53. doi: 10.1089/thy.1995.5.249, PMID: 7488863

[53] Wiesner A Gajewska D Paśko P. Levothyroxine interactions with food and dietary supplements-a systematic review. Pharmaceuticals. (2021) 14:206. doi: 10.3390/ph14030206, PMID: 33801406 PMC8002057

[54] Bilezikian JP Loeb JN Gammon DE. Induction of sustained hyperthyroidism and hypothyroidism in the Turkey: physiological and biochemical observations. Poult Sci. (1980) 59:628–34. doi: 10.3382/ps.0590628

