# Supplementary material for: Case series: effects of an induced molting protocol using levothyroxine in five captive banded penguins (genus Spheniscus)
Source: Front Vet Sci. 2025 Sep 10;12:1544599. doi: 10.3389/fvets.2025.1544599 (PMC12458642; doi:10.3389/fvets.2025.1544599)
Supplement: Supplementary file 1 [file Data_Sheet_1.pdf]

CARE case report guidelines

CARE Checklist of information to include when writing a case report

(cc) BY-NC-ND

Topic	Item	Checklist item description	Reported on Line
**Title**	1	The diagnosis or intervention of primary focus followed by the words "case report"	V
**Key Words**	2	2 to 5 key words that identify diagnoses or interventions in this case report, including "case report"	V
**Abstract (no references)**	3a	Introduction: What is unique about this case and what does it add to the scientific literature?	V
	3b	Main symptoms and/or important clinical findings	V
	3c	The main diagnoses, therapeutic interventions, and outcomes	V
	3d	Conclusion—What is the main "take-away" lesson(s) from this case?	V
**Introduction**	4	One or two paragraphs summarizing why this case is unique (**may include references**)	V
**Patient Information**	5a	De-identified patient specific information.	V
	5b	Primary concerns and symptoms of the patient.	V
	5c	Medical, family, and psycho-social history including relevant genetic information	V
	5d	Relevant past interventions with outcomes	V
**Clinical Findings**	6	Describe significant physical examination (PE) and important clinical findings.	V
**Timeline**	7	Historical and current information from this episode of care organized as a timeline	V
**Diagnostic Assessment**	8a	Diagnostic testing (such as PE, laboratory testing, imaging, surveys).	V
	8b	Diagnostic challenges (such as access to testing, financial, or cultural)	V
	8c	Diagnosis (including other diagnoses considered)	V
	8d	Prognosis (such as staging in oncology) where applicable	V
**Therapeutic Intervention**	9a	Types of therapeutic intervention (such as pharmacologic, surgical, preventive, self-care)	V
	9b	Administration of therapeutic intervention (such as dosage, strength, duration)	V
	9c	Changes in therapeutic intervention (with rationale)	V
**Follow-up and Outcomes**	10a	Clinician and patient-assessed outcomes (if available)	V
	10b	Important follow-up diagnostic and other test results	V
	10c	Intervention adherence and tolerability (How was this assessed?)	by aquarists
	10d	Adverse and unanticipated events	V
**Discussion**	11a	A scientific discussion of the strengths AND limitations associated with this case report	V
	11b	Discussion of the relevant medical literature **with references**.	V
	11c	The scientific rationale for any conclusions (including assessment of possible causes)	V
	11d	The primary "take-away" lessons of this case report (without references) in a one paragraph conclusion	V
**Patient Perspective**	12	The patient should share their perspective in one to two paragraphs on the treatment(s) they received	Not applicable
**Informed Consent**	13	Did the patient give informed consent? Please provide if requested	Yes ☐ No ☑
